# Supplementary material for: Fecal Microbiota Transplantation Controls Murine Chronic Intestinal Inflammation by Modulating Immune Cell Functions and Gut Microbiota Composition
Source: Cells. 2019 May 28;8(6):517. doi: 10.3390/cells8060517 (PMC6628315; doi:10.3390/cells8060517)
Supplement: Supplementary file 1 [file cells-08-00517-s001.pdf]

Supplementary Material

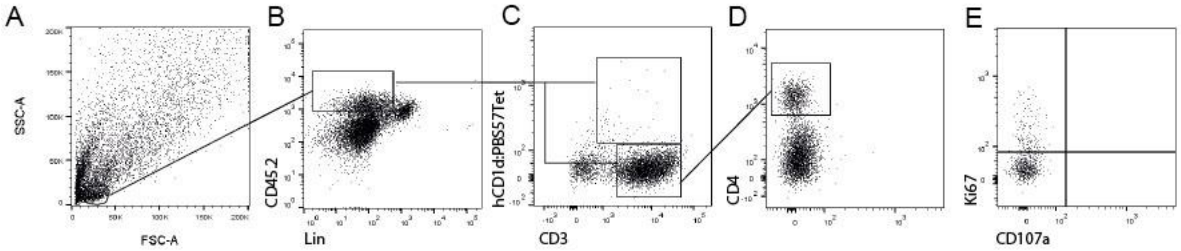

Figure S1. Gating strategy. (**A**) Forward and side scatter of colonic LPMC gate indicates living lymphocytes. (**B**) Epithelial cell-excluding gate based on CD45.2 expression and Lineage (CD19, CD11c, CD11b)-excluding gate. (**C**) The mCD1d:PBS57 Tet and CD3 staining to identify iNKT cells. (**D**) On CD3+ cells, CD4 (or CD8) staining cells to identify ConvT cells. (**E**) Ki67 and CD107a on iNKT, CD4+, or CD8+ cells.

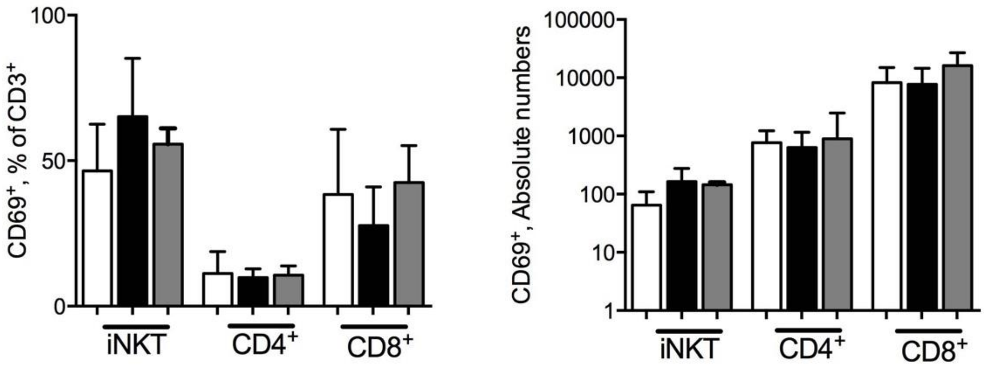

Figure S2. *CD69* expression Frequencies and absolute numbers of CD69 positive colonic CD4^{+} T, CD8^{+} T cells, and iNKT cells in untreated (white bars), chronic- DSS-treated (black bars), and FMT-treated (grey bars) mice 63 days after starting DSS administration.

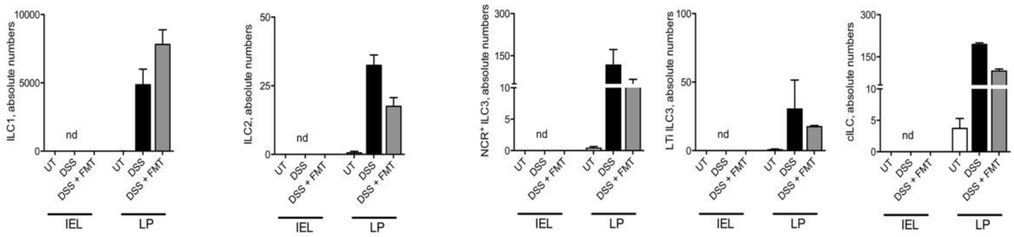

Figure S3. Analysis of innate lymphoid cells (ILC). (**A**) Lamina propria (LP) and intra-epithelial (IEL) ILC1, ILC2, ILC3 (NCR, LTi, cILC) absolute numbers from chronic DSS-treated mice (untreated, white

bars; DSS/TNBS/Chronic DSS, black bars; FMT, grey bars). Significance was determined using unpaired two-tailed Mann-Whitney test and expressed as mean±SEM. Outliers were detected with Grubb's test. $P < 0.05$ (*) was regarded as statistically significant.

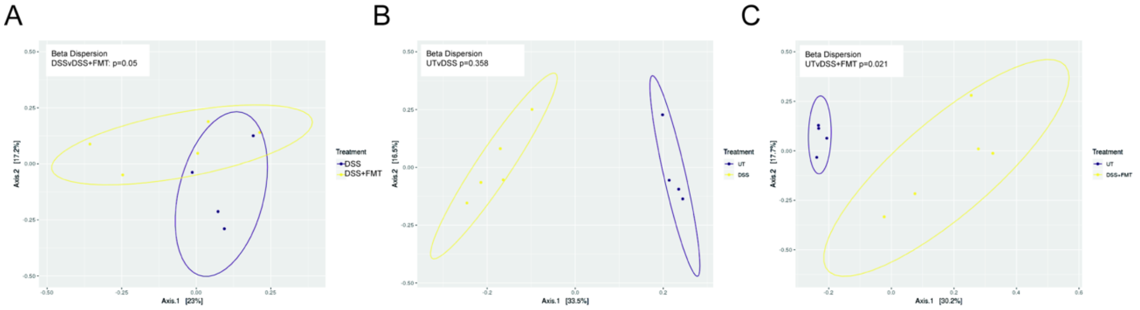

Figure S4. Microbiome clustering based on Principal Coordinate Analysis (PCoA) of unweighted UniFrac distances of faecal gut microbiota derived from (**A**) cDSS- and cDSS + FMT-, (**B**) UT and cDSS- (blue dots), and (**C**) UT and cDSS+ FMT-treated mice. Significant differences in beta-diversity between groups were determined by beta-dispersion and PERMANOVA with p-value correction by Bonferonni (padj), with $P < 0.05$ as cut-off for significance.

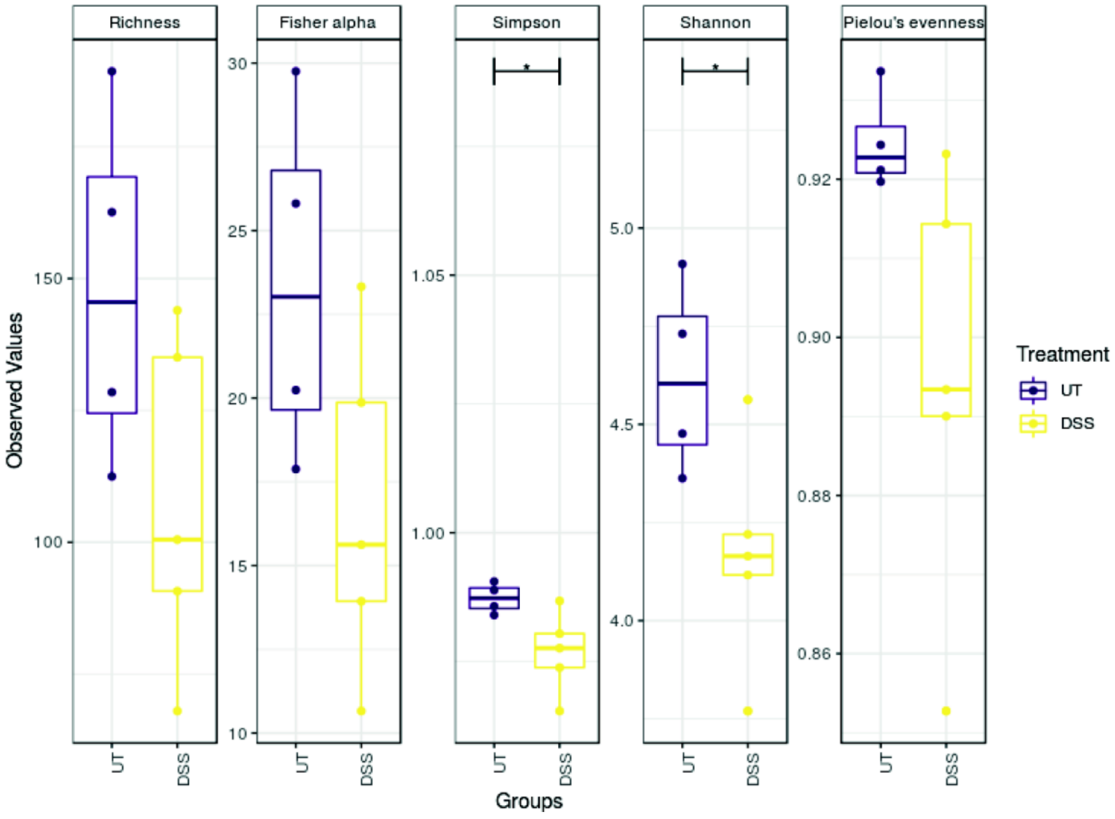

Figure S5. Comparison of alpha-diversity metrics between untreated (violet boxes) and cDSS (yellow boxes)-treated mice. Significant differences in alpha-diversity between groups were determined by ANOVA, with $P < 0.05$ as cut-off for significance.

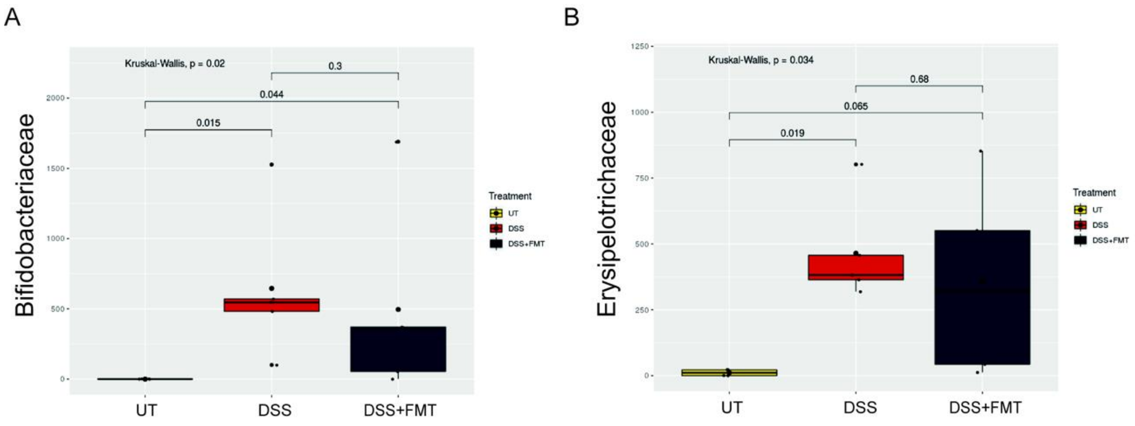

Figure S6. Abundance comparison of (**A**) *Bifidobacteriaceae* and (**B**) *Erysipelotrichaceae* families and in untreated (yellow box), cDSS- (red box), and cDSS + FMT (violet box)-treated mice. Significant differences in taxon abundance between groups were determined by Kruskal-Wallis, with $P < 0.05$ as cut-off for significance.

Table S1. Scoring scheme for evaluation of intestinal inflammation.

	**Criterion**	**Definition**	**Score Value**
**Inflammatory Cell Infiltrate**	Severity (leukocyte density of lamina propria area infiltrated in evaluated hpf)	No infiltrate	0
		Minimal acute (<10%)	0.25
		Mild chronic (10–25%, scattered neutrophils)	0.5
		Moderate chronic (26–50%)	0.75
		Marked (>51%, dense infiltrate)	1
	Extent (expansion of leukocyte infiltration)	Mucosal	0.5
		Mucosal and submucosal	0.75
**Epithelial Changes**	Hyperplasia (increase in epithelial cell numbers in longitudinal crypts, visible as crypt elongation)	No hyperplasia	0
		Minimal (<25%)	0.25
		Mild (26–35%)	0.5
		Moderate (36–50%, mitoses in the upper third of the crypt epithelium)	0.75
		Marked (>51%, mitoses in crypt epithelium distant from crypt base)	1
	Goblet cell loss (reduction of goblet cell numbers relative to baseline goblet cell numbers per crypt)	No loss	0
		Minimal (<25%)	0.25
		Mild (26–35%)	0.5
		Moderate (36–50%)	0.75
		Marked (>51%)	1
**Mucosal architecture**	Ulceration (epithelial defect reaching beyond muscolaris mucosae)	No ulcers	0
		Ulcers	0.25
	Granulation tissue (connective tissue repair with new capillaries, surrounded by infiltrating cells, hypertrophied areas)	No granulation tissue	0
		Granulation tissue	0.25
	Mucosal thickness and crypt depth	No thickening	0

		Thickening	0.5
	Glandular rarefaction	No rarefaction	0
		Rarefaction	0.5
	Dysplasia	No dysplasia	0
		Dysplasia	0.5
		MAX SCORE	6

Table S2. Primer sequences.

Primer	Product	Vendor
IL17a	Qiagen (QuantiTect)	QT00103278
IFNg	Qiagen (QuantiTect)	QT01038821
Rpl32	Qiagen (QuantiTect)	QT00131992
Camp1	Qiagen (QuantiTect)	QT00241003
S100A8	Qiagen (QuantiTect)	QT00250264
IL1b	Qiagen (QuantiTect)	QT010483555

Table S2. *Cont.*

Primer	Forward	Reverse	bp	Vendor
TNF	TCTTCTCATTCCTGCTTG	CACTTGGTGGTTTGCT	200	SIGMA
IL6	CTCTGGGAAATCGTGGA	GCAAGTGCATCATCG	77	SIGMA
Muc1	TACCCTACCTACCACACTCACG	CTGCTACTGCCATTACCTGC	95	SIGMA
Muc3	CTTCCAGCCTTCCCTAAACC	TCCACAGATCCATGCAAAAC	119	SIGMA
Muc4	GAGAGTTCCCTGGCTGTGTC	GGACATGGGTGTCTGTGTTG	101	SIGMA

Table S3. FACS antibodies and dyes.

Mouse	clone	Vendor
CD45.2	104	Biolegend
CD3	17A2	BD
CD4	GK1.5	BD
CD11c	HL3	BD
CD19	1D3	BD
CD11b	M1/70	BD
F4/80	BM8	Biolegend
Ly6g	1A8	Biolegend
Ly6c	AL-21	eBioscience
MHC-II	M5/114.15.2	eBioscience
Ki67	16A8	Biolegend
CD107a	1D4B	BD
Ter119	TER-119	BD
TCR α/β	H57.597	BD
TCR γ/δ	UC713B5	Biolegend
CD90	53-2.1	BD
CD117	2B8	BD
CD127	A7R34	Biolegend
Nkp46	29A1.4	BD
RorγT	AFKJS9	eBioscience
ST-2	DIH9	Biolegend
CD25	PC61	BD
IFNg	XMG1.2	BD
IL17A	TC11-18H10.1	Biolegend
IL13	eBio13A	eBioscience
Zombie Dye		Biolegend (Cat #423104)

Zo-1	ZO1-1A12	Invitrogen
